# Favorable clinical outcome of recurrent deep chest wall infection caused by pan-resistant *Klebsiella pneumoniae* treated with cefotaxime plus polymyxin B: a case report

**DOI:** 10.1590/0037-8682-0088-2026

**Published:** 2026-07-17

**Authors:** Sophia Aléo Bomfim, José Eduardo Souza Echeverria, Ruana Carolina Cabral da Silva, Andyane Freitas Tetila, Andressa Leite Ferraz de Melo, Gleyce Hellen de Almeida de Souza, Cleide Adriane Signor Tirloni, Simone Simionatto

**Affiliations:** 1 Universidade Federal da Grande Dourados, Laboratório de Pesquisa em Ciências da Saúde, Dourados, MS, Brasil.; 2 Universidade Federal da Grande Dourados, Hospital Universitário HUBrasil, Dourados, MS, Brasil.

**Keywords:** Antimicrobial resistance, Pan-resistant *K. pneumoniae*, Cefotaxime, Polymyxin B, Case report, Synergism

## Abstract

This case report describes a 43-year-old man admitted in November 2024 with recurrent deep anterior chest wall and thoracoabdominal soft-tissue infection caused by *Klebsiella pneumoniae*. Multidrug-resistant *K. pneumoniae* was isolated in December 2024. From January to April 2025, the patient required prolonged hospitalization, repeated debridements, and skin grafting, complicated by flap infection, acute kidney injury, and ICU admission. After initial improvement with polymyxin B and tigecycline, a severe recurrent infection caused by pan-resistant *K. pneumoniae* occurred in June 2025. Given the limited therapeutic options, treatment was changed to cefotaxime plus polymyxin B, leading to complete clinical recovery, which was later confirmed by *in vitro* synergy results.

## INTRODUCTION

Infections caused by multidrug-resistant *Klebsiella pneumoniae* are a growing concern, particularly when associated with carbapenemase production, which severely limits treatment options^1^ ^,^ ^2^. The emergence of pan-resistant microorganisms resistant to all available antimicrobial classes further complicates clinical management and is often associated with adverse outcomes^3^ ^,^ ^4^.

A comprehensive literature search was conducted in the Cochrane Library, LILACS, SciELO, MEDLINE, PubMed, and PubMed Central (PMC) databases using the following keywords “*Klebsiella pneumoniae*,” “Pan-resistant,” “synergism,” “Polymyxin B,” and “Cefotaxime.” A total of 5,702 articles were identified; however, after screening, only six were included, all with *in vitro* analyses and two also incorporating *in vivo* murine models. Notably, no clinical studies or case reports evaluating this combination therapy were identified. Although multidrug-resistant *K. pneumoniae* has been extensively investigated, reports specifically describing pan-resistant isolates treated with cefotaxime plus polymyxin B, particularly with documented *in vitro* synergism and corresponding clinical evolution, remain scarce. This gap underscores the clinical relevance of the present case and its contribution to the existing evidence.

This study was approved by the Research Ethics Committee of the Federal University of Grande Dourados (protocol 4.014.325/2020).

## CASE REPORT

In November 2024, a 43-year-old man was admitted to a public hospital in Dourados, Mato Grosso do Sul. His medical history included type 2 diabetes mellitus, prior ischemic stroke, and right lower limb amputation following trauma, and he reported substantial weight loss before hospitalization. He presented with extensive infectious soft tissue damage to the anterior chest wall, reportedly following a spider bite in October 2024. The patient worked in recyclable waste collection, which may increase exposure to arthropods. The spider was not collected at the time of the incident and was not identified; therefore, the etiology was based solely on the patient’s report. Empirical antibiotic therapy was initiated with ceftriaxone (2 g every 24 h) combined with clindamycin (900 mg every 8 h).

Because of expansion of the necrotic area and persistent fever spikes, the regimen was increased to piperacillin/tazobactam (4.5 g every 6 h) combined with vancomycin (loading dose, 25-30 mg/kg, followed by 15-20 mg/kg every 12 h, adjusted according to renal function and serum levels), indicating therapeutic failure of the initial regimen. Vancomycin was subsequently replaced with linezolid (600 mg every 12 h), while piperacillin/tazobactam (4.5 g every 6 h) was maintained. The patient remained on this regimen for 34 days. In December 2024, multidrug-resistant *K. pneumoniae* was isolated from chest wall tissue samples after prolonged broad-spectrum antibiotic therapy, which was associated with selective pressure and lesion chronicity. The strain was resistant to carbapenems, cephalosporins, fluoroquinolones, aminoglycosides, and combinations with β-lactamase inhibitors, and was susceptible only to polymyxin B.

In January 2025, the infectious disease service identified progression to a pan-resistant bacterial profile. During the three-month hospitalization, clinical and surgical management included multiple debridements, skin grafting, thoracic reconstruction, and negative pressure therapy. The patient’s condition worsened due to flap infection and acute kidney injury, requiring dialysis and intensive care. During this period, multidrug-resistant *K. pneumoniae* infection was treated with polymyxin B (approximately 20,000-27,000 IU/kg, with dose adjustments for nephrotoxicity) from January 3 to April 6, 2025, and tigecycline (100 mg every 12 h) from January 7 to March 7, 2025. In March 2025, deep tissue cultures identified a carbapenem-resistant *K. pneumoniae* isolate that remained susceptible to polymyxin B. Combination therapy with polymyxin B and tigecycline was maintained within the treatment periods described above. After six weeks of antimicrobial therapy and clinical improvement, the patient was discharged on April 11, 2025.

The patient was readmitted to the same public hospital in Dourados on June 1 after developing fever and worsening local infection. Piperacillin-tazobactam (4.5 g every 6 h) was initiated. During negative pressure wound therapy, the abdominal wound drained serohemorrhagic fluid. Laboratory findings revealed anemia (hemoglobin, 7.6-8.2 g/dL), mild leukocytosis, reactive thrombocytosis, and an initial C-reactive protein level of 149 mg/L, which gradually declined. Serum creatinine ranged from 1.2 to 1.4 mg/dL. Because of infection and lack of surgical source control, empirical antimicrobial therapy with meropenem (1 g every 8 h) was initiated. Owing to the absence of clinical response, therapy was empirically escalated to polymyxin B (loading dose, 25,000 IU/kg, followed by 15,000 IU/kg every 12 h), combined with tigecycline (loading dose: 100 mg, followed by 50 mg every 12 h), administered from June 3 to July 7, 2025.

On June 3, pan-resistant *K. pneumoniae* was isolated from abscess and wound secretions, showing resistance to broad-spectrum cephalosporins (cefepime and ceftazidime), β-lactamase inhibitor combinations (piperacillin-tazobactam), carbapenems (meropenem and imipenem), aminoglycosides (amikacin and gentamicin), fluoroquinolones (ciprofloxacin and levofloxacin), and sulfonamides (trimethoprim-sulfamethoxazole). Resistance to polymyxin B (minimum inhibitory concentration [MIC] = 64 µg/mL) and ceftazidime-avibactam (MIC >8/4 µg/mL) was also observed.

On June 4, antimicrobial therapy was changed to polymyxin B (15,000 IU/kg every 12 h, corresponding to approximately 30,000 IU/kg/day) combined with cefotaxime (2 g every 6 h), optimized according to pharmacokinetic/pharmacodynamic principles. After this change, the patient showed progressive clinical improvement, remained afebrile and hemodynamically stable, and had gradual reductions in C-reactive protein levels and purulent drainage during ongoing surgical and wound care.

The pan-resistant *K. pneumoniae* isolate was evaluated using checkerboard microdilution to assess *in vitro* synergy for ceftibuten-polymyxin B, cefotaxime-meropenem, cefotaxime-ceftibuten, and meropenem-polymyxin B. Synergism was defined as a fractional inhibitory concentration index (FICI) ≤ 0.5; assays were performed in duplicate. Cefotaxime-polymyxin B exhibited the strongest synergistic interaction, reducing the polymyxin MIC from 64 to 4 µg/mL and yielding combined MICs of 4/4 µg/mL. Conversely, ceftibuten-polymyxin B achieved a less pronounced effect (combined MICs, 8/8 µg/mL). No relevant interaction was observed for cefotaxime-meropenem or meropenem-polymyxin B, as combined MICs remained >64/64 µg/mL. Resistance genes were assessed using polymerase chain reaction with specific primers targeting *bla*_KPC_, *bla*_NDM_, and *bla*_OXA-48_ carbapenemase genes and the plasmid-mediated colistin resistance *mcr-1* gene (Table 1).

**TABLE 1:** Timeline of antimicrobial therapies and major clinical events.

Period (date)	Hospital/unit	Clinical condition/procedure	Antimicrobials (dose)	Comments
Oct 2024 to Dec 2024	Hospital 1 (Dourados)	Extensive lesion after bite; local wound care and procedures	Initial therapy not detailed in the transferred medical record	-
Jan 2025 to Apr 2025	Hospital 2 (hospitalization)	Surgical debridements, skin grafting, abdominal flap. Complications including flap infection and acute kidney injury	Polymyxin B (approx. 20,000-27,000 IU/kg; dose adjusted due to nephrotoxicity) + tigecycline (100 mg every 12 h) + piperacillin-tazobactam (4.5 g every 6 h)	Deep tissue culture positive for *K. pneumoniae* susceptible to polymyxin B
Apr 11, 2025	Discharge	Clinical improvement	-	Persistent thoracic fistula with minimal drainage
May 26, 2025	Primary health unit	Fever, purulent drainage, infection recurrence	Piperacillin-tazobactam (4.5 g every 6 h)	Referred to Hospital 2
Jun 01, 2025	Hospital 2 (readmission)	Extensive thoracic wound, sternal fistula; abdominal negative-pressure wound therapy	Meropenem (1 g every 8 h)	No clinical response
Early Jun 2025	Hospital 2	Initial therapeutic failure	Polymyxin B (25,000 IU/kg loading dose; 15,000 IU/kg every 12 h) + tigecycline (100 mg loading dose; 50 mg every 12 h)	Empirical regimen
Jun 03, 2025	Hospital 2	Isolation of pan-resistant *K. pneumoniae* (BD Phoenix)	Regimen adjusted to polymyxin B (15,000 IU/kg every 12 h) + cefotaxime (2 g every 8 h)	Clinical improvement and hospital discharge

## DISCUSSION

Pan-resistant *K. pneumoniae* infections are among the most challenging scenarios in contemporary clinical practice, particularly in patients with extensive wounds, prolonged hospitalization, and multiple surgical interventions. These conditions favor the emergence and selection of multidrug-resistant strains, leading to limited therapeutic options and frequently unfavorable outcomes. This case report describes a severe infection requiring complex clinical management, in which a pan-resistant phenotype emerged after antimicrobial exposure, including loss of susceptibility to polymyxin B. Although reduced polymyxin susceptibility after prolonged therapy has been previously reported, it is commonly associated with sustained selective pressure, persistent biofilm formation, and compromised tissue barriers^5^. Notably, combination therapy was followed by clinical improvement, and *in vitro* analyses demonstrated synergistic antimicrobial activity of the administered regimen, providing microbiological support for the therapeutic strategy.

Limited effective therapeutic options create a challenging clinical context. In severe infections with difficult-to-control foci, antimicrobial combinations are frequently used in routine practice despite limited evidence for many regimens^6^. Currently, ceftazidime/avibactam is considered a key therapeutic option for infections caused by carbapenem-resistant *K. pneumoniae*, particularly KPC-producing strains^1^. However, availability may be restricted in some healthcare settings, and therapeutic challenges persist in cases with pan-resistance profiles. From a microbiological standpoint, *in vitro* synergy testing can serve as a supplementary tool to explore interactions between agents, generating laboratory data that may be considered alongside clinical, surgical, and stewardship factors, without implying a direct causal relationship with patient outcomes.

The synergism between polymyxins and carbapenems or tigecycline has been reported^7^. However, evidence involving third-generation cephalosporins remains scarce^8^. In this case, the polymyxin MIC was reduced to 4 µg/mL in the presence of cefotaxime, indicating enhanced *in vitro* activity of the combination under the tested conditions. This finding is consistent with the hypothesis that specific “nonconventional” pairs may exhibit beneficial interactions even when individual agents have limited activity^9^. As a single-case observation, these data should be interpreted descriptively, without assuming that the *in vitro* findings directly determined the therapeutic response.

This case report describes a favorable in-hospital clinical course in a patient with pan-resistant *K. pneumoniae* infection. The identification of *bla*_KPC_ confirmed significant carbapenem resistance. Conversely, the absence of the plasmid-mediated *mcr-1* gene, together with the ambiguous underpinnings of the elevated polymyxin MIC, suggests alternative resistance mechanisms. In settings with limited access to novel antimicrobials, documenting microbiological interaction profiles may inform evaluation of rescue strategies and generate hypotheses, without implying efficacy beyond the observed outcomes^10^.

These results provide real-world microbiological data on a pan-resistant *K. pneumoniae* isolate, including molecular characterization and *in vitro* synergy testing, along with a detailed description of the patient’s clinical course. However, given the absence of pharmacokinetic/pharmacodynamic modeling and the inherent limitations of single-case studies, causal inferences regarding treatment effects are not feasible^11^. Nevertheless, the observed *in vitro* synergism for cefotaxime-polymyxin B may be reported as a parallel laboratory finding to inform future research on antimicrobial combinations in extreme resistance settings^12^. Thus, the favorable clinical outcome in this case should be interpreted in the context of combined therapeutic strategies, including optimized antimicrobial therapy.

## Data Availability

Research data are available in the body of the article.
